# Femtosecond Infrared Spectroscopy Resolving the Multiplicity of High-Spin Crossover States in Transition Metal Iron Complexes

**DOI:** 10.1021/jacs.4c01637

**Published:** 2024-03-23

**Authors:** Clark Zahn, Mariachiara Pastore, J. Luis Perez Lustres, Philippe C. Gros, Stefan Haacke, Karsten Heyne

**Affiliations:** † Department of Physics, Free University Berlin, Arnimallee 14, D-14195 Berlin, Germany; ‡ Université de Lorraine, CNRS, LPCT, F-54000 Nancy, France; § Université de Lorraine, CNRS, L2CM, F-54000 Nancy, France; ∥ Université de Strasbourg—CNRS, IPCMS, 67034 Strasbourg, France

## Abstract

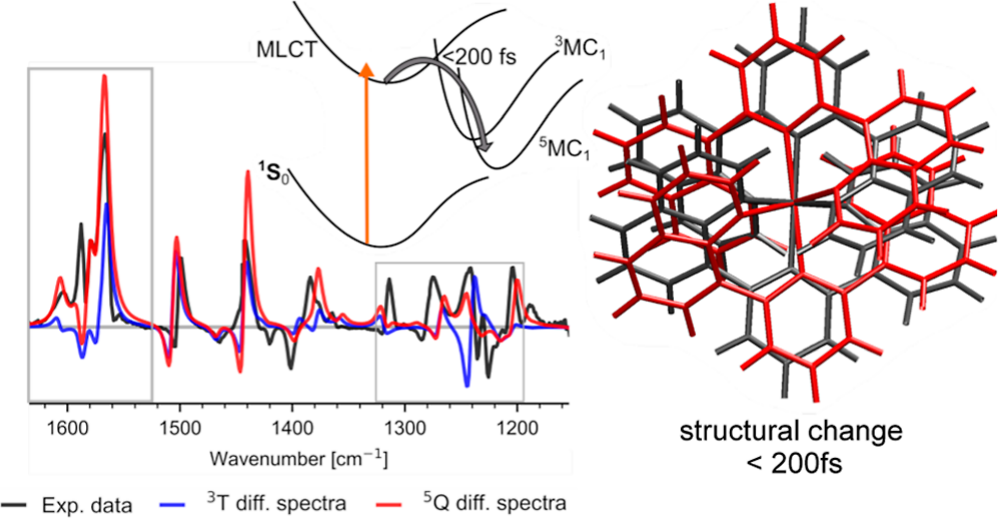

Tuning the photophysical properties of iron-based transition-metal complexes is crucial for their employment as photosensitizers in solar energy conversion. For the optimization of these new complexes, a detailed understanding of the excited-state deactivation paths is necessary. Here, we report femtosecond transient mid-IR spectroscopy data on a recently developed octahedral ligand-field enhancing [Fe(dqp)_2_]^2+^ (**C1**) complex with dqp = 2,6-diquinolylpyridine and prototypical [Fe(bpy)_3_]^2+^ (**C0**). By combining mid-IR spectroscopy with quantum chemical DFT calculations, we propose a method for disentangling the ^5^Q_1_ and ^3^T_1_ multiplicities of the long-lived metal-centered (MC) states, applicable to a variety of metal–organic iron complexes. Our results for **C0** align well with the established assignment toward the ^5^Q_1_, validating our approach. For **C1**, we find that deactivation of the initially excited metal-to-ligand charge-transfer state leads to a population of a long-lived MC ^5^Q_1_ state. Analysis of transient changes in the mid-IR shows an ultrafast sub 200 fs rearrangement of ligand geometry for both complexes, accompanying the MLCT → MC deactivation. This confirms that the flexibility in the ligand sphere supports the stabilization of high spin states and plays a crucial role in the MLCT lifetime of metal–organic iron complexes.

## Introduction

**Figure 1:**
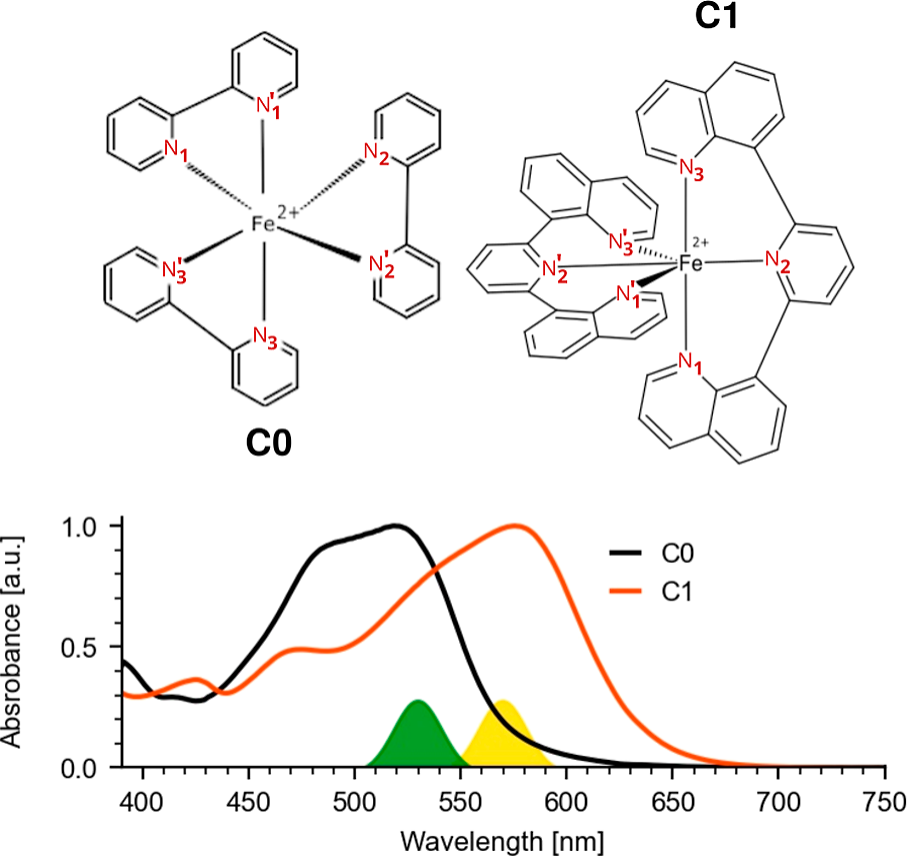
Chemical structure of **C0** and **C1** (upper panel). Labeling of the coordinating nitrogen atoms highlighted in red color (the counteranion was PF_6_ in both cases). Normalized visible absorption spectrum of the two complexes **C0** and **C1** (lower panel). Spectral positions of the visible excitation pulses at wavelengths 530 nm (**C0**, green-filled curve) and 570 nm (**C1**, yellow-filled curve) are indicated.

Transition-metal complexes are a class of photosensitizers with versatile applications in many fields including solar energy conversion.^1−4^ However, many promising complexes rely on scarce, expensive, and toxic central metals, such as ruthenium or iridium.^5,6^ Thus, recent advancements brought forth new iron-based transition-metal complexes.^7−23^ However, for many newly synthesized iron compounds, including different Fe(II) polypyridyl complexes, the initial excited metal-to-ligand charge-transfer (MLCT) state is deactivated into a high spin metal-centered (MC) state on a subpicosecond timescale.^19,24−26^ Yet, a long MLCT lifetime is mandatory for employing iron transition metal complexes as efficient photosensitizers. An important parameter for tuning the MLCT lifetime is the ligand-field splitting.^19,27,28^ Indeed, enhancement of the ligand-field splitting can lead to destabilization of the high-spin MC state, thus prolonging the MLCT lifetime. One way to achieve this is by promoting an octahedral coordination of the central metal, which reduces the angular strain on the metal center. We recently tried to achieve this with a [Fe(dqp)_2_]^2+^ (**C1**) complex with dqp = 2,6-diquinolylpyridine (see Figure 1 upper panel), accomplishing an almost ideal octahedral ligand-field enhancing geometry.^19^ However, upon excitation of **C1**, the MLCT state was found to relax on a sub-picosecond timescale into a low-energy MC state, which we tentatively assigned to the quintet MC state (^5^Q_1_). Unfortunately, the UV/VIS data did not allow for a clear differentiation between ^5^Q_1_ and ^3^T_1_. In previous work on different Fe(II) polypyridyl complexes, the assignment of the long-lived MC state and possible intermediates proved to be challenging, requiring extensive research and expensive methods.^29^ In this regard, proto-typical [Fe(bpy)_3_]^2+^ (**C0**) (see Figure 1 upper panel) was established as a well-investigated benchmark system^25,26,30−38^ for Fe(II) transition-metal complexes.

Here, we report on the femtosecond transient mid-IR spectroscopy study of both **C0** and **C1**, covering the ligand fingerprint region in the spectral range from 1620 to 1180 cm^–1^. Even though it suffers from a lower time resolution compared to that of the spectroscopy study in the visible or UV range, infrared spectroscopy is particularly sensitive to structural changes, making it well suited for the investigation of MLCT and MC states and potentially distinguish between various spin multiplicities. However, few reports of femtosecond infrared spectroscopy on transition-metal complexes are available.^30,39−41^ Particularly, for the ligand fingerprint region, no in-depth investigation of the long-lived MC states has been reported. We excited the sample to an MLCT state, and within our time resolution, we traced relaxation dynamics in the MC state. We show for the first time that the combination of experimental results and DFT IR spectra calculations allows for a direct assignment of the multiplicity of the observed long-lived MC states.

## Materials and Methods

### Sample Preparation

[Fe(bpy)_3_]^2+^ (**C0**) and [Fe(dqp)_2_]^2+^ (**C1**) with dqp = 2,6-diquinolylpyridine were prepared according to refs (19 and 42), respectively; the counteranion was PF_6_ in both cases. Both samples were dissolved in deuterated acetonitrile and prepared in sample cells with a thickness of 100 μm with an absorption of 0.7–0.9 OD at 530 and 570 nm, respectively.

### Transient Absorption

Femtosecond laser pulses were generated starting from pulses delivered by a commercially available 1.088 kHz Ti/Sa laser system (Coherent Legend USP, 80 fs pulses at 808 nm). Spectrally tunable visible pump pulses were generated by a home-built two-stage NOPA generating output pulses of ∼15 μJ. An AOPDF pulse shaper (Fastlite, Dazzler) compresses the output pulses to (60 ± 20) fs. A λ/2-plate was used to set the polarization of the pump-beam, alternating between perpendicular and parallel pump–probe configuration. The isotropic signal was obtained from parallel and perpendicular signals, as follows: *A*_iso_ = (*A*_∥_ + 2*A*_⊥_)/3. Here, we show isotropic signals. Before exciting the sample, the pump-pulses were attenuated to 0.2–0.5 μJ. The diameter of the pump beam on the sample was (180 ± 40) μm (fwhm). IR probe beams with an energy of 50 nJ were generated, as reported elsewhere.^43^ The generated IR beam was split into two reflections, one acting as the probe beam and the other as a reference. Both beams passed through the same sample volume, with the reference pulse arriving 1.5 ns before the probe, reducing the shot-to-shot signal-to-noise ratio.^44^ Both IR pulses are detected by dispersing the beams with an imaging spectrograph and recording both beams simultaneously with a 128 × 128 element MCT-array (2DMCT from PhaseTech). The spectral resolution was better than 3 cm^–1^. The system response duration and time-zero position were determined using a germanium wafer, yielding Δ*t* = (170 ± 50) fs. The sample was moved with a Lissajous scanner to ensure a fresh sample volume between consecutive pump pulses. The probe pulses were delayed by using a mechanical translation stage.

### Data Analysis

The data analysis was performed in Python using the skultrafast^44,45^ package.

### Computational Methods

For the quantum mechanical calculations, we used the level of theory validated in a previous work reported by some of us^19^ and summarized in the following. Geometry optimization without any symmetry constraint and frequency calculations for the singlet ground state (^1^S_0_) and the triplet (^3^T_1_) and quintet (^5^Q_1_) excited states were carried out by DFT calculations employing the B3LYP exchange and correlation functional and a 6-31+G(d,p) basis set; empirical Grimme’s D3 corrections were also applied.^46^ The unrestricted Kohn–Sham formalism was adopted for the open-shell electronic solutions. Solvent effects (acetonitrile) were modeled by using the polarizable continuum model, as implemented in the Gaussian 16 suite of programs.^47^

## Results and Discussion

Normalized steady-state visible absorption spectra of **C0** and **C1** are shown in Figure 1, lower panel. Both complexes display dominant broad visible absorption bands ranging from 430 to 570 nm (**C0**, black line) and 440 to 650 nm (**C1**, orange line).

**Figure 2:**
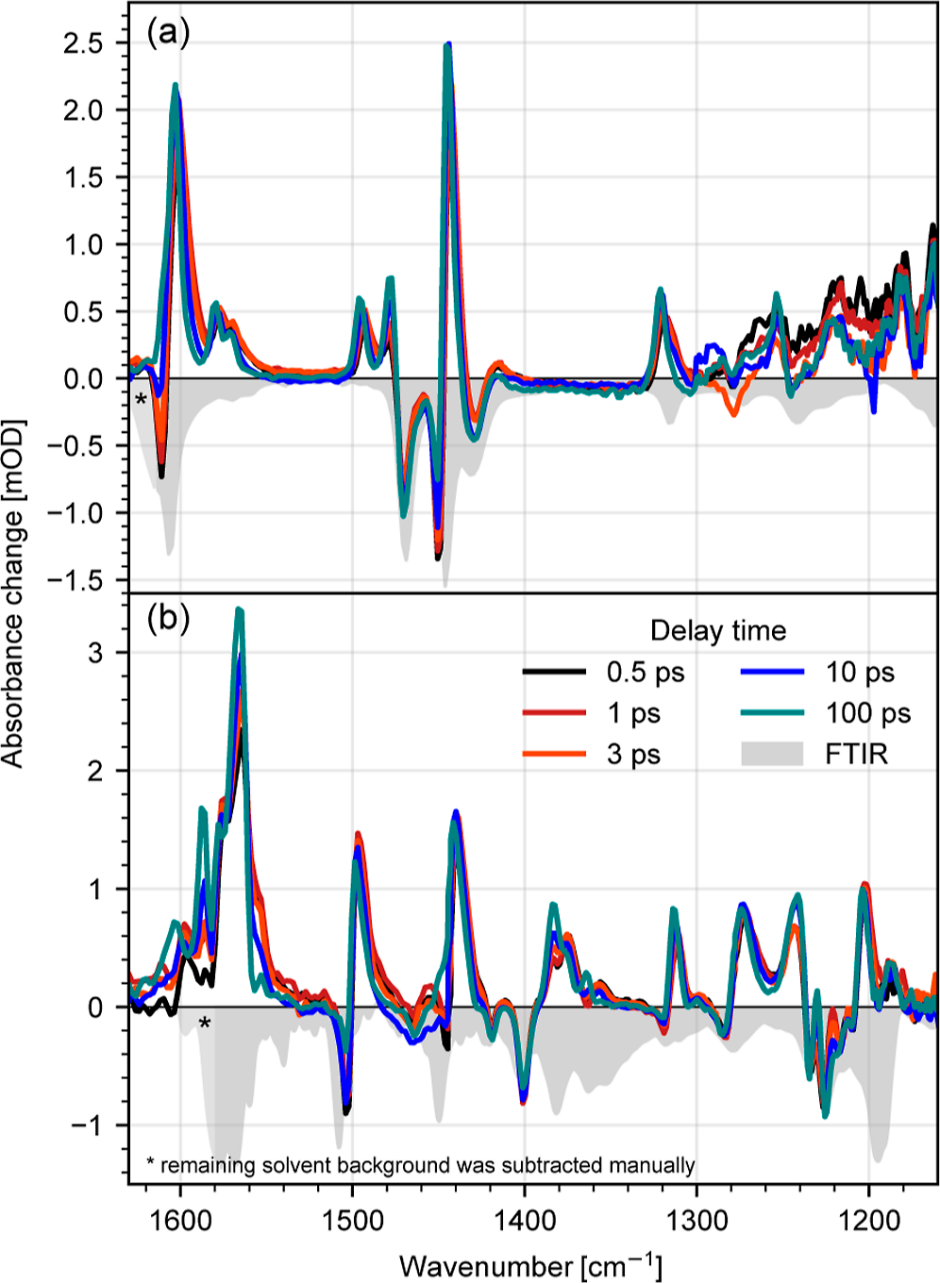
Transient visible pump–mid-IR probe spectra of the complexes **C0** (a) and **C1** (b) for selected delay times. The ground-state absorption spectrum is indicated by the inverted and scaled FTIR spectra (light gray) for both complexes. The color code for selected delay times is identical for **C0** (a) and **C1** (b).

Transient mid-IR absorption data of the two complexes **C0** and **C1** are shown in Figure 2a,b, respectively. Positive contributions are associated with excited-state absorption (ESA), while negative signal shows bleaching of the respective ground-state vibration. The complexes were excited around their visible absorption maxima at 530 nm (**C0**) and 570 nm (**C1**), as indicated in Figure 1. Upon excitation, both complexes show strong spectral changes in the fingerprint region from 1150 to 1630 cm^–1^, attributed to structural reorganization of the ligands. Bands in the range from 1600 to 1400 cm^–1^ are associated with different combinations of ν(CN) and ν(CC) stretching modes, while the lower energetic region down to 1150 cm^–1^ shows changes in the ring deformation and C–H bending modes.

Analysis of the polarization-resolved signal shows no significant anisotropy for both **C0** and **C1** after the system response in the MC state (see Supporting Information Figure S1). This is most likely attributed to a loss of the initial photoselection due to the high symmetry of the ligands or delocalization of the MLCT state. This is in line with previous work on [Ru(bpy)_3_]^2+^, reporting a loss of anisotropy on a timescale of a few hundred femtoseconds.^48^

### Vibrational Dynamics

The MLCT to MC transition is reported to happen on a timescale of sub-50 fs^26^ and ∼450 fs (upon excitation at 400 nm)^19^ for **C0** and **C1**, respectively. For **C0**, this is out of the range of our system response of (170 ± 50) fs. For **C1**, we find no ultrafast dynamics on a 0.4–1 ps timescale, suggesting that excitation at 570 nm leads to faster MLCT → MC deactivation. Inspection of the early sub-300 fs dynamics at around 1430 cm^–1^ indicates an early evolution of the signal on a timescale of 200–250 fs (see Supporting Information Figure S3), suggesting MLCT → MC deactivation on that timescale. Unfortunately, this is not resolvable with our time resolution. Supplementary vis-pump–vis-probe measurements reveal a MLCT → MC deactivation with (110 ± 10) fs excited at 550 nm (see Supporting Information Figure S4). Thus, we conclude that the earliest spectra resolved in the mid-IR experiment reported here already correspond to the MC manifold.

Following the MLCT → MC transition, both complexes **C0** and **C1** show significantly stronger ESA signals compared to bleaching signals. This demonstrates an increase of oscillator strength for the vibrational modes in the excited states compared with that in the ground state. This is rather unusual since molecules such as corroles^49^ or chlorophyll a^50^ exhibit similar vibrational signal strengths in the electronic excited state compared to its ground state. Compared to these photoactive systems, the binding forces between iron and ligands in **C0** and **C1** are rather weak, so that the higher flexibility of the complexes enables an ultrafast rearrangement of ligand geometry, changing bond distances, angles, and dipole moments. Thus, we assign the observed increase of oscillator strength in the electronic excited state to a structural rearrangement of the ligands in combination with a dipole moment change due to partial charge redistribution. These changes occur faster than our time resolution of (170 ± 50) fs, showing that the structural rearrangements happen on a sub-200 fs timescale, in the MLCT state or concomitant with the MLCT → MC conversion.

**Figure 3:**
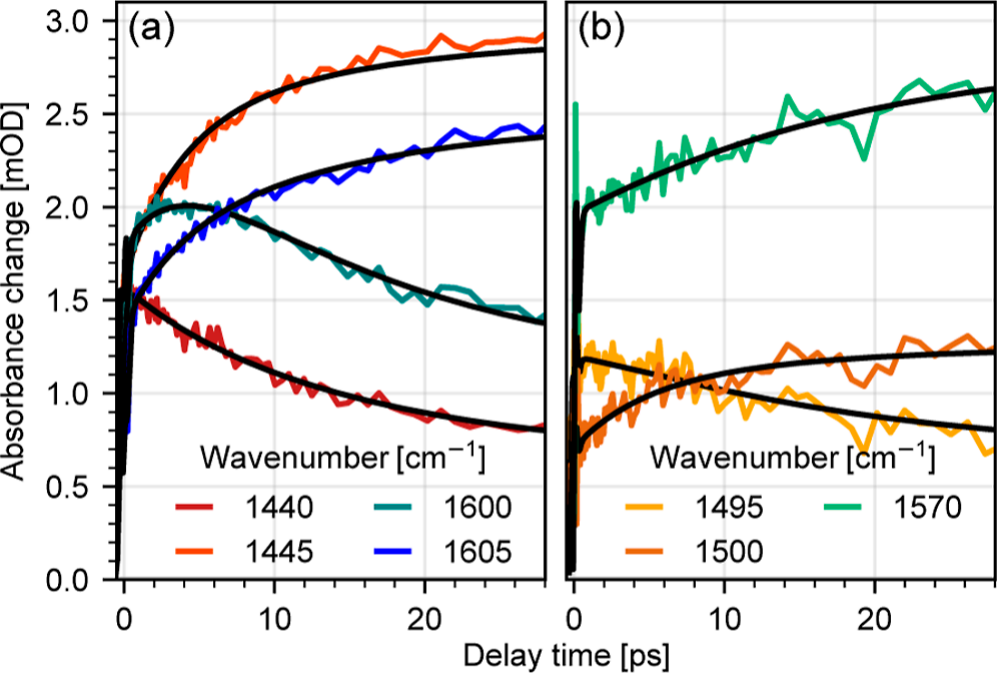
Transient traces for **C0** (a) and **C1** (b) at selected spectral positions. Black lines show modeling of the data, obtained from global fitting with two exponential decay functions and a constant term.

**Figure 4:**
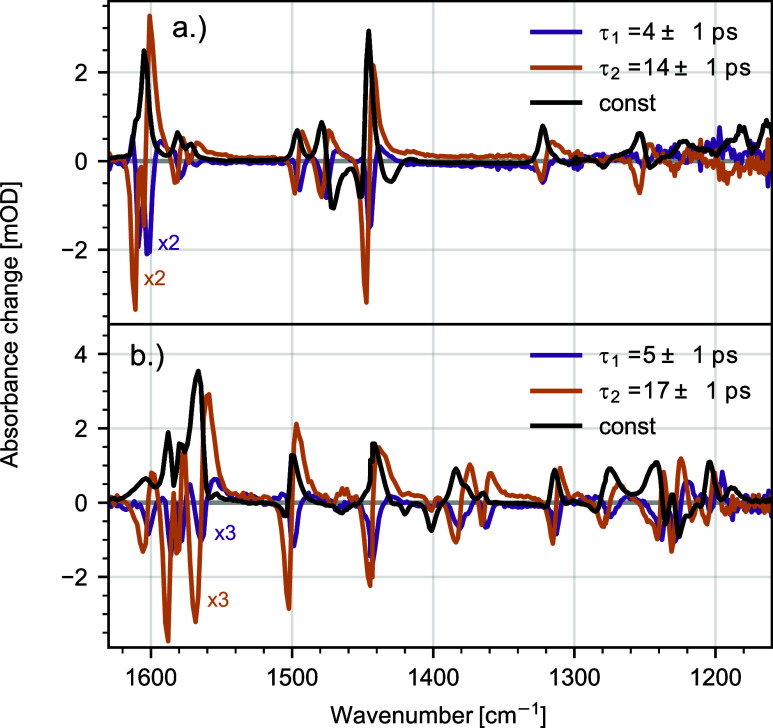
Mid-IR DAS of **C0** (a) and **C1** (b) obtained from global fitting with two exponential decays. DAS for both **C0** and **C1** (purple and orange lines) are attributed to fast intramolecular energy redistribution and vibrational cooling. For better visibility, DAS of **C0** and **C1** are multiplied by a factor of 2 and 3, respectively.

Transient changes on the picosecond timescale for selected wavenumbers are shown in Figure 3a,b. After 30 ps, no relevant changes in the excited state are observed in the investigated time window ranging up to 150 ps. For both complexes, we observe a 2-fold evolution of the signal, with a short time constant of a few picoseconds and a second slower component with 10–20 ps (see selected transients, Figure 3). Thus, we modeled the spectral dynamics with global analysis using a model of two exponential decay functions  and a constant *c*(ν). In the case of **C0**, this yields values of τ_1_ = (4 ± 1) ps and τ_2_ = (14 ± 1) ps. Corresponding decay associated spectra (DAS) showing the amplitudes  are displayed in Figure 4a. Inspection of the DAS values for τ_1_ and τ_2_ exhibits a series of positive and negative peaks. This pattern of positive and negative peaks reflects a blue shift and narrowing of the peaks, well visible in the decrease of signal at 1440 and 1600 cm^–1^ and associated increase of signal at 1445 and 1605 cm^–1^ (see Figure 3a).

These spectral features are typical for intramolecular redistribution of excess energy and energy relaxation associated with dissipating the energy to low-frequency modes.^51^ Thus, we assign both the short time constant of τ_1_ = (4 ± 1) ps and the second time constant of τ_2_ = (14 ± 1) ps to dissipation of excess energy to low-frequency modes and subsequent cooling of the low-frequency modes in the MC state.

**Figure 5:**
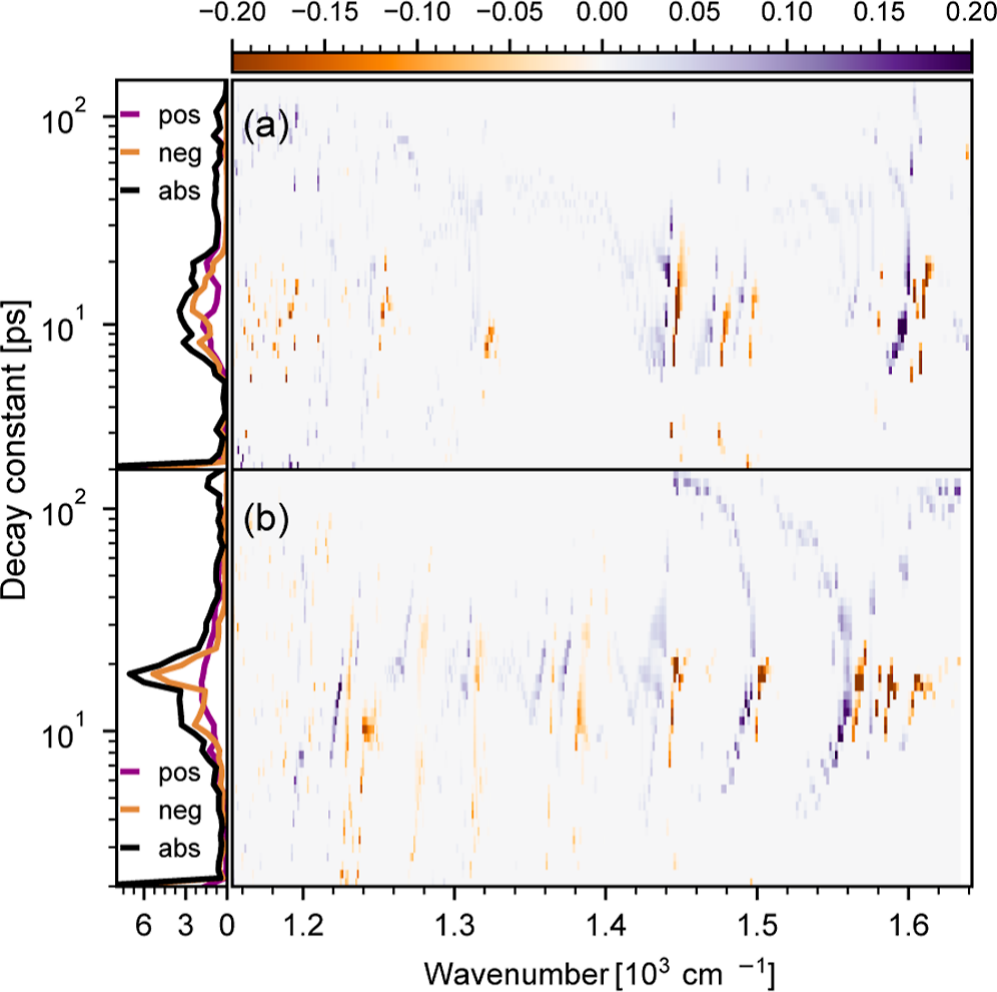
LTDMs of **C0** (a) and **C1** (b). LTDA was performed with a set of 50 logarithmically distributed time points τ_*i*_. On a timescale of 10–20 ps, both complexes exhibit pairs of negative and slightly blue-shifted positive contributions; with increasing decay times, the pairs shift to higher frequencies indicating red shift and cooling of vibrations; positive decaying signals show a stronger distribution of decay times due to narrowing of the vibrational bands. These dynamics are attributed to energy redistribution and vibrational relaxation.

Comparing our results to previous studies, the fast decay time of (4 ± 1) ps agrees well with the observed time constant of 3.4 ps by Auböck and Chergui,^26^ while other studies using femtosecond X-ray absorption near edge structure (XANES) and ultrafast electron diffraction (UED) observe faster time constants of (1.1 ± 0.2) ps (XANES),^33^ 1.6 ps (XANES),^34^ and (2.4 ± 0.4)ps (UED).^37^ For the slower component of τ_2_ = (14 ± 1 ps, the observed dynamics are not within the measured delay times of both XANES studies^33,34^ and are not detected in the UED study.^37^ On the other hand, prior work of Smeigh et al.^30^ using femtosecond-stimulated Raman scattering reveals a slow component of (10 ± 3) ps but missing a fast component. Another study using femtosecond transient absorption by Miller and McCusker^36^ reports that vibrational cooling is observed with ∼5–10 ps. However, these small discrepancies in the obtained time constants are not very surprising. Global analysis using a simple exponential model is generally not sufficient to model complex cooling dynamics.^52,53^ Particularly, cooling-induced blue shifts and narrowing of bands are not well modeled.^53^ Therefore, we performed a lifetime density analysis (LTDA),^44,45^ fitting a series of *i* fixed exponentials  to the data set. Corresponding lifetime density maps (LTDMs) for **C0** and **C1** applying LTDA with a set of 50 logarithmically distributed time points τ_*i*_ are shown in Figure 5a,b, respectively.

Negative and positive amplitudes, *A*_*i*_(ν), are displayed in orange and purple, respectively. Sum of all contributions for the whole spectral range is displayed in the left panel of the figure. Inspection of the LTDM reveals the complex nature of the underlying processes. In line with the DAS, adjacent diagonal positive and negative contributions reflect a blue shift and narrowing of the bands. On the other hand, LTDA reveals the nonexponential behavior of the vibrational relaxation dynamics, well visible in the smeared out contributions ranging between 10 and 20 ps, as depicted left in the panel of Figure 5a.

After energy redistribution and vibrational relaxation, the complex is in the thermally equilibrated MC state. The associated spectrum is represented by the constant contribution of the DAS (black line in Figure 4a).

For **C1**, global analysis yields time constants of τ_1_ = (5 ± 1) ps and τ_2_ = (17 ± 1) ps. Associated DAS are depicted in Figure 4b, while the results of LTDA and associated LTDM are presented in Figure 5b. The results reveal very similar vibrational relaxation dynamics for **C1** as for **C0**. In short, after initial population of the MLCT state, ultrafast ISC leads to deactivation of the MLCT and population of the MC state accompanied by structural rearrangement. This is the first state resolved in the IR transient absorption data. Afterwards, redistribution of excess energy to low-frequency modes leads to shifting and narrowing of the positive signals, well visible in the decrease of signal at 1495 cm^–1^ and increase of signal at 1500 cm^–1^ (Figure 3b). LTDA highlights the nonexponential behavior of the vibrational relaxation dynamics, revealing vibrational relaxation dynamics occurring between 10 and 20 ps. The remaining signal is attributed to the thermally equilibrated MC state, represented by the constant DAS of **C1** (see the black line in Figure 4b).

### Coherent Oscillations

**Figure 6:**
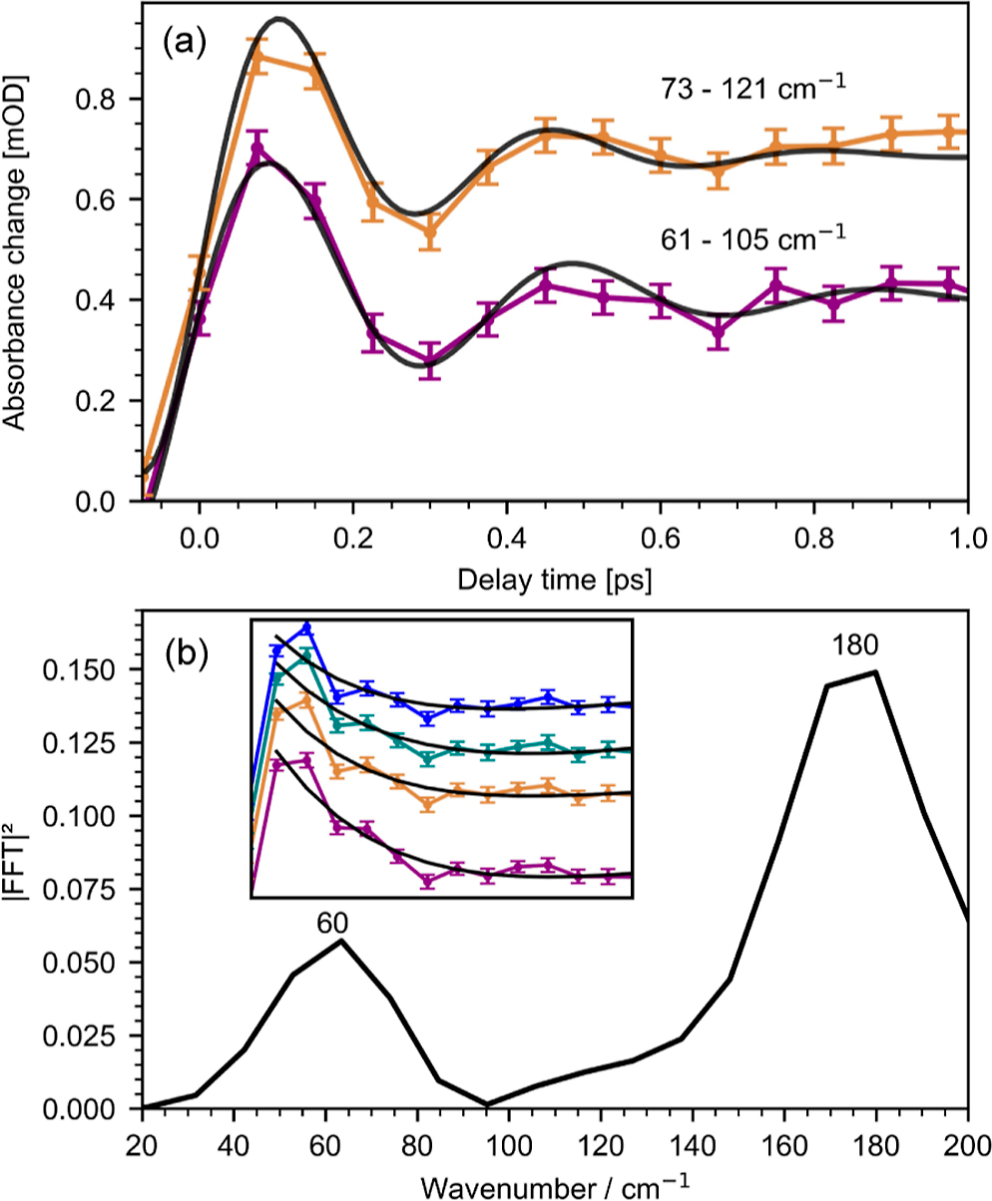
(a) Transient traces of **C0** revealing coherent oscillations with frequencies of 73–121 cm^–1^ (at 1593 cm^–1^, orange line) and 61–105 cm^–1^ (at 1589 cm^–1^, purple line). Damping is modeled with (300 ± 100) fs (at 1589 cm^–1^) and (200 ± 100) fs (at 1593 cm^–1^). (b) Fourier analysis of the residuals after fitting a biexponential decay to selected transients at 1430–1436 cm^–1^ of **C1** (inset). Fourier analysis reveals dominant oscillating features at 150–200 cm^–1^ and small contributions at 40–80 cm^–1^.

Closer inspection of the transient mid-IR absorption of the ν(CN) and ν(CC) stretching bands at 1570–1610 cm^–1^ of **C0** reveals coherent oscillations. Transient traces of selected wavenumbers are depicted in Figure 6a. In order to characterize the coherent motion, we modeled the data using an exponentially decaying cosine function convoluted with a Gaussian function representing the instrument response function. This yields frequencies of 73–121 cm^–1^ and 61–105 cm^–1^, with damping times of (300 ± 100) fs and (200 ± 100) fs, at 1589 and 1593 cm^–1^, respectively. We assign the coherent oscillation to the formation of a vibrational wave packet in the ^5^Q_1_ state, changing the Fe–N coordination. Our results agree well with previous work of Auböck and Chergui^26^ (Vis TA), Lemke et al.^34^ (XANES), and Zang et al.^54^ (femtosecond X-ray fluorescence spectroscopy), all three studies showing clear coherent oscillations attributed to the breathing modes of the Fe–N coordination sphere.

For **C1**, inspection of the transient mid-IR absorption at around 1430–1440 cm^–1^ shows small coherent oscillation close to our S/N level and experimental time resolution. However, Fourier analysis of the residuals indicates an oscillating feature with a frequency of 150–200 cm^–1^ (see Figure 6b). This matches well with the low-frequency modes modeled by our quantum chemical calculations, at 145, 197, 199, and 202 cm^–1^. Atomic displacements of the low-frequency modes are shown in Supporting Information Figures S7 and S8. All four modes are associated with vibrations in the Fe–N coordination sphere, similar to that of **C0**. The Fourier analysis also shows contributions at 40–80 cm^–1^, in line with the results reported by Darari et al.,^19^ showing a clear coherent oscillation with 53–65 cm^–1^. Here, our quantum chemical calculations give three possible associated low-frequency modes at 66, 70, and 82 cm^–1^. All of these modes show clear ligand deformation, affecting the Fe–N coordination sphere.

### Multiplicity of the MC State

**Figure 7:**
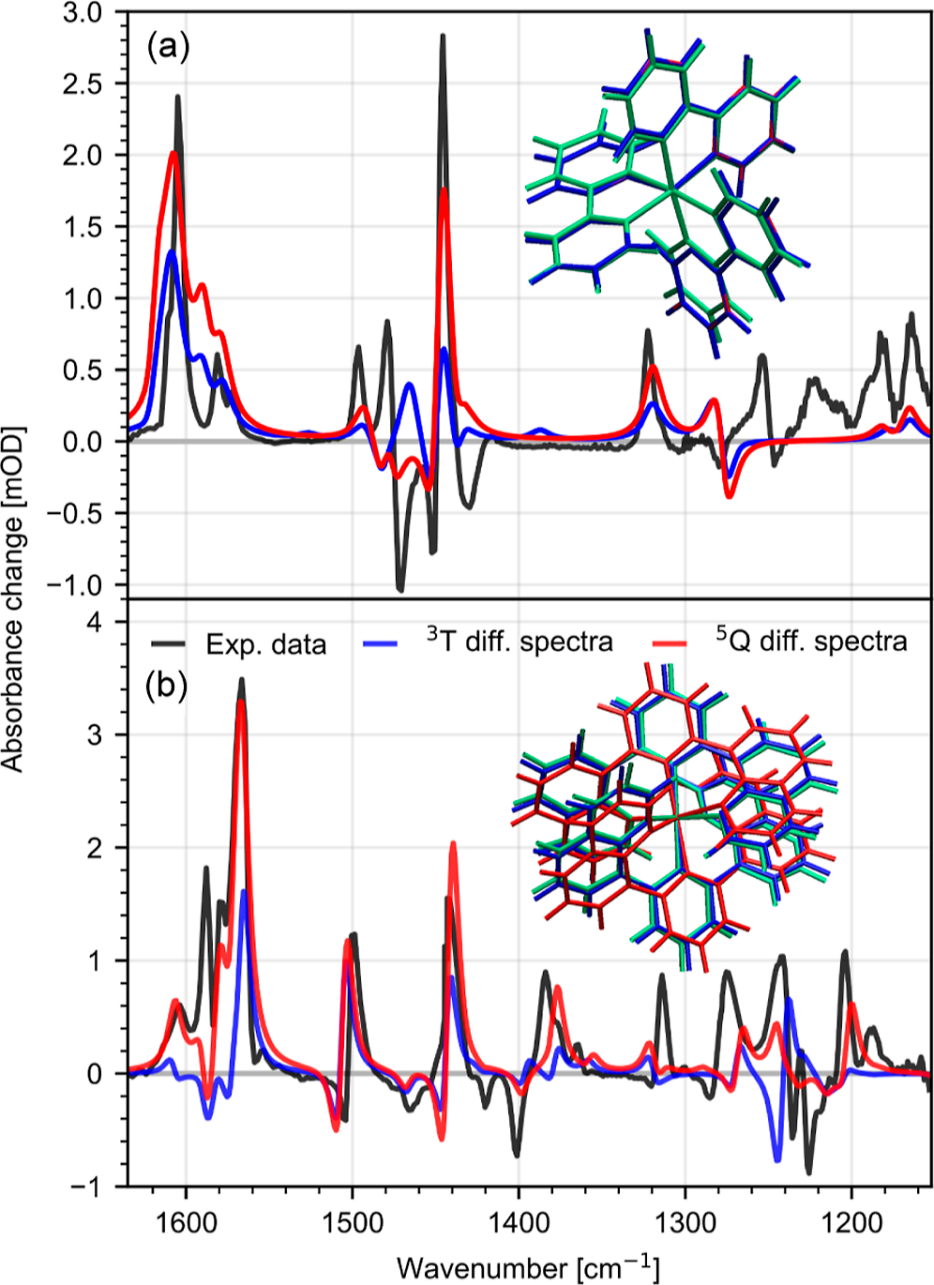
Experimental difference spectra (black) of the thermally equilibrated MC state of **C0** (a) and **C1** (b). Theoretical spectra are obtained using a Lorentzian convolution with a width of 5 cm^–1^ and a vibrational frequency scaling factor of 0.98, matching the experimental and theoretical spectra. Theoretical difference spectra are calculated for the triplet MC ^3^T_1_ (^3^T_1_ – ^1^S_0_) (blue) and quintet MC ^5^Q_1_ (^5^Q_1_ – ^1^S_0_) (red). Inset: stick representation of the geometry-optimized structures of **C0** and **C1** for the ground-state ^1^S_0_ (green), ^3^T_1_ state (blue), and ^5^Q_1_ state (red). **C0** shows very little structural difference between ^3^T_1_ and ^5^Q_1_ configurations.

In order to determine the multiplicity of the long-lived MC state in **C0** and **C1** and to distinguish between the ^3^T_1_ and ^5^Q_1_ states, we performed quantum chemical calculations. From the calculations, we obtain the respective ^1^S_0_, ^3^T_1_, and ^5^Q_1_ state infrared spectra and compare the theoretical results to the experimental data. For a detailed analysis of the individual spectra, see the Supporting Information (Figure S6 and Tables S1 and S2). Geometry-optimized structures for the ground-state ^1^S_0_ (green), ^3^T_1_ state (blue), and ^5^Q_1_ state (red) for both complexes **C0** and **C1** are presented in Figure 7a,b insets, respectively.

Optimized structures for **C0** show that both the ^3^T_1_ and ^5^Q_1_ states exhibit different geometries with longer Fe–N distances compared to the ^1^S_0_ state (see Table 1). This is expected as these are antibonding states. On the other hand, the two high spin states ^3^T_1_ and ^5^Q_1_ have a very similar geometries with only subtle differences. Thus, the two high spin states cannot be easily distinguished, as shown in Figure 7a.

**Table 1:** Fe–N Distances (in Å) from Geometry-Optimized Structures of the ^1^S_0_, ^3^T_1_, and ^5^Q_1_ States^a^

Fe–N	^1^S_0_	^3^T_1_	^5^Q_1_
**C0**			
Fe–N1	2.000	2.170	2.198
Fe–N2	2.000	2.148	2.163
Fe–N3	2.000	1.985	2.178
Fe–N1′	2.000	1.993	2.168
Fe–N2′	2.000	2.300	2.190
Fe–N3′	2.000	2.085	2.173
**C1**			
Fe–N1	2.024	2.160	2.191
Fe–N2	2.000	1.980	2.160
Fe–N3	2.025	2.161	2.198
Fe–N1′	2.025	2.160	2.191
Fe–N2′	2.000	1.980	2.160
Fe–N3′	2.025	2.161	2.198

a The atom labeling is reported in Figure 1.

For **C1**, all three states have clearly different geometries. These changes in the ligand orientation directly impact the coordination spheres surrounding the central iron atom, affecting the Fe–N distance. Changes in the Fe–N distance for both complexes **C0** and **C1** are presented in Table 1. These structural differences, particularly the lengthening of the iron-ligand bond, lead to a change in the force constants, particularly for the N–C stretching vibrations. These changes in force constants have a direct impact on the normal modes of the complexes. We performed normal-mode analysis and determined the associated infrared spectra for the respective geometry. In order to compare the theoretical results with the experimental data, we calculated the theoretical difference spectra of ^3^T_1_ (^3^T_1_ – ^1^S_0_) and ^5^Q_1_ (^5^Q_1_ – ^1^S_0_), as shown in Figure 7. Experimental MC difference spectra of the thermally equilibrated MC state are shown in black. By comparing the calculated difference spectra of **C0** for multiplicities ^3^T_1_ (blue line) and ^5^Q_1_ (red line), we observe clear differences at around 1450 cm^–1^ as well as minor differences at around 1600 cm^–1^. Comparison with the experimental data (black line) shows that the experimental spectrum agrees better with the ^5^Q_1_ difference spectrum than with the ^3^T_1_ difference spectrum. This is best visible in the region between 1440 and 1520 cm^–1^. Further, at 1480 cm^–1^, the experimental data show a clear negative bleaching signal, matching the negative signal of the ^5^Q_1_ difference spectrum. In contrast, the difference spectrum associated with ^3^T_1_ shows a positive contribution. In general, for the spectral region between 1440 and 1520 cm^–1^, we find that even though the individual band intensities are different, the ^5^Q_1_ difference spectrum matches the experimental data noticeably better than the ^3^T_1_ difference spectrum.

In the case of **C1** (Figure 7b), comparison of the calculated difference spectra of ^3^T_1_ (blue line) and ^5^Q_1_ (red line) reveal clear differences at around 1600 cm^–1^ and between 1200 and 1300 cm^–1^, indicating spin-sensitive marker bands at 1570–1620 cm^–1^ and 1200 cm^–1^. Particularly, the modes at around 1600 cm^–1^, associated with C–N stretching vibrations, are expected to be very sensitive to the difference in geometry between ^3^T_1_ and ^5^Q_1_. Considering the experimental data, we find that the ^5^Q_1_ difference spectrum agrees much better with the experimental MC spectrum than the ^3^T_1_ difference spectrum. For example, the positive signal at 1570–1620 cm^–1^ is well represented in the ^5^Q_1_ difference spectrum, while it is absent in the ^3^T_1_ difference spectrum. Likewise, the two positive peaks at 1240 and 1280 cm^–1^ agree well with the ^5^Q_1_ difference spectrum, whereas the ^3^T_1_ difference spectrum shows an opposite negative signal at 1240 cm^–1^. This allowed us to assign the long-lived component of **C1** to the ^5^Q_1_ state.

However, for **C0**, the picture is not as clear since the striking differences are limited to a small spectral region, as discussed above. Thus, in addition to the inspection of individual bands, we determined the overlap between the experimental and the calculated spectra in order to quantify the agreement. For this, we multiplied the experimental signal *A*_exp_ with the signal of the respective calculated difference spectra *A*_theo_ for both spin states ^3^T_1_ and ^5^Q_1_ and averaged it over the whole spectral region. This yields an overlap factor *f*
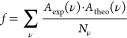
1where *N*_ν_ is the number of data points. The larger the value of *f*, the better the agreement. The calculated overlap factors for both complexes are **C0**: (^3^T_1_:0.041)/(^5^Q_1_:0.088) and **C1**: (^3^T_1_:0.036)/(^5^Q_1_:0.157). The results show that for **C0**, the overlap of ^5^Q_1_ with the experimental data is twice that of ^3^T_1_, and for **C1**, the overlap of the ^5^Q_1_ is even four times the overlap factor of ^3^T_1_. With this, we assign the long-lived component of **C0** to the ^5^Q_1_ state. This is consistent with the established assignment.^25,30,32,34^ For **C1**, the clearly better overlap of experimental and calculated spectra corroborates our initial assignment toward the ^5^Q_1_ state.

### Electron Densities of ^3^T_1_ and ^5^Q_1_

**Figure 8:**
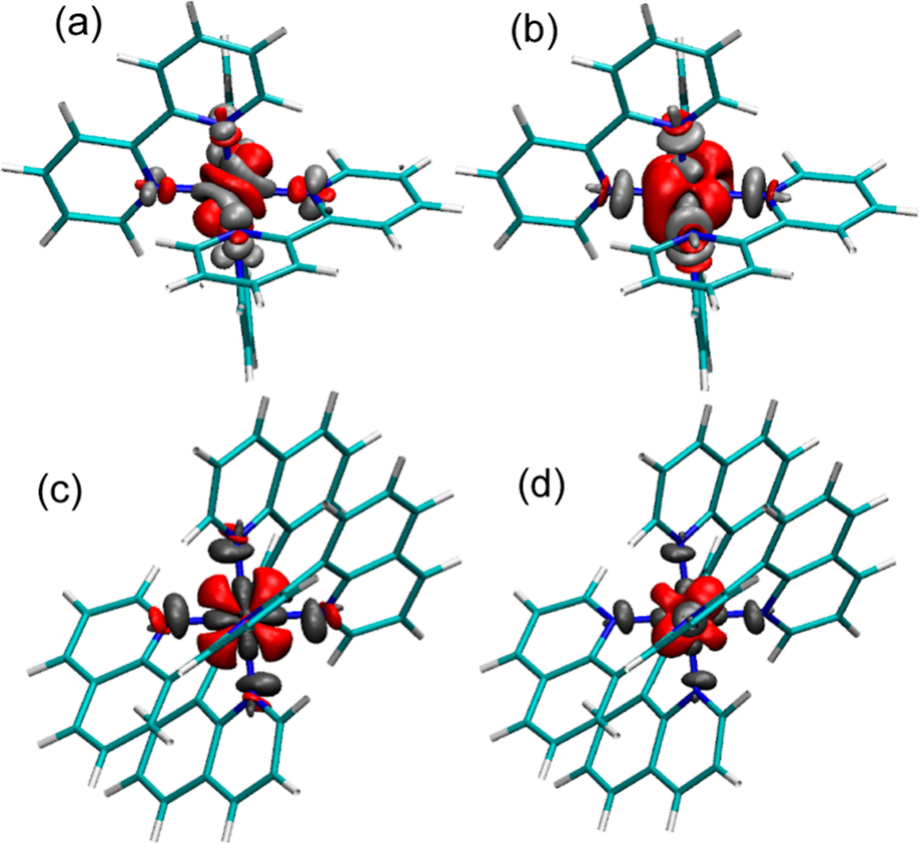
Change in the electron density for ^1^S_0_ → ^3^T_1_ [left column: **C0** (a) and **C1** (c)] and ^1^S_0_ → ^5^Q_1_ [(right column: **C0** (b) and **C1** (d)] transition calculated from quantum chemical simulations. Dark gray color is associated with charge accumulation, while red color shows charge depletion.

The MC character of the ^3^T_1_ and ^5^Q_1_ states becomes apparent in the changes in the electron density for the ^1^S_0_ → ^3^T_1_ and ^1^S_0_ → ^5^Q_1_ transition obtained from B3LYP electronic structure calculations, as depicted in Figure 8. Dark gray color is associated with charge accumulation, while red color shows charge depletion. For **C0** (see Figure 8a,b), we find clear differences in electron density at the central iron atom between ^3^T_1_ and ^5^Q_1_. On the other hand, changes at the coordinating nitrogen atoms are very similar for ^3^T_1_ and ^5^Q_1_. Contrarily, for **C1** (see Figure 8c,d), the differences in electron density between ^3^T_1_ and ^5^Q_1_ at the central iron atom are less pronounced, while the coordinating nitrogen atoms are more affected. We find that for the ^3^T_1_ state, the axial nitrogen atoms N2 and N2′ exhibit almost no changes, while the radial coordinating nitrogen atoms N1, N1′ and N3, N3′ show an accumulation of charge at the nitrogen atoms. On the other hand, for the ^5^Q_1_ state, we see a charge accumulation at all six nitrogen atoms. We assume that the differences in electron densities are likely attributed to the asymmetry in the coordination sphere, where the N2 and N2′ are two single pyridine rings, while the N1, N1′ and N3, N3′ belong to quinolines. These differences in charge accumulation are clearly reflected in the calculated equilibrium Fe–N bond distance (see Table 1). For the ^3^T_1_ state, we observe an increase of bond length for N1–Fe, N1′–Fe and N3–Fe, N3′–Fe, while the bond length of the axial nitrogens N2–Fe and N2′–Fe remains almost unchanged; however, for the ^5^Q_1_ state, the bond length increases for all six nitrogen bonds.

## Conclusions

Here, we demonstrate that ultrafast mid-IR spectroscopy supported by state-of-the-art quantum chemical calculations allows for a clear assignment of transient spin multiplicities of MC states in iron transition-metal complexes. For **C0**, which is a benchmark system for iron transition-metal complexes, we show that despite the subtle differences between ^3^T_1_ and ^5^Q_1_ state equilibrium geometries, the comparison of experimental with calculated difference spectra of the ^3^T_1_ and ^5^Q_1_ states allows a clear assignment toward the ^5^Q_1_ state, in agreement with previous reports.^25,30^ For **C1**, we report a first unambiguous assignment of the long-lived MC state, ascribing it to ^5^Q_1_. In the case of **C1**, the quantum chemical calculations exhibit differences in axial electron densities between the ^3^T_1_ state and the ^5^Q_1_ state, altering transition dipole moments of vibrational modes and their frequencies. This gives rise to clear spin-sensitive marker bands at 1570–1620 cm^–1^ and 1200 cm^–1^. Differences in the axial electron densities are likely attributed to asymmetry in the coordination sphere of **C1**. Here, the axial coordination of N2 and N2′ is given by two single pyridine rings, while the other 4 coordinating Ns′ belong to quinolines.

Besides the determination of the multiplicities of the long-lived MC state, investigation of the transient mid-IR dynamics provides direct insight into structural changes and tracing of energy redistribution and relaxation of excess energy processes. The presented infrared data for both complexes **C0** and **C1** reveal an increase of oscillator strength in the excited state on a sub-200 fs timescale. We attribute this change in oscillator strength to rearrangement of the ligand geometry with deviating bond lengths and partial charge distribution, accompanying the MLCT → MC deactivation. This suggests that the flexibility in the ligand sphere allows for the stabilization of high spin states and decreases the MLCT lifetime. Our results thus support the conclusion of Darari and co-workers,^19^ suggesting that a higher flexibility in the ligand sphere compromises the possible effects of enhanced ligand-field splitting, stabilizing the MC state and shortening the MLCT lifetime. In addition, this aligns well with reports of significantly increased MLCT lifetimes of 528 ps^14^ and even up to nanoseconds^22^ for more rigid complexes.

Inspection of the early dynamics of **C0** and **C1** shows coherent oscillations of the breathing modes of the Fe–N coordination sphere. For **C0**, we find oscillations with 61–121 cm^–1^, in line with previous studies,^26,34,54^ while for **C1**, we find small coherent oscillations yielding frequencies of 40–80 cm^–1^ and 150–200 cm^–1^, matching possible low-frequency modes of our quantum chemical calculations. Moreover, transient mid-IR spectra allow for the direct observation of vibrational relaxation dynamics. Even though **C0** was well investigated by applying different kinds of state-of-the-art spectroscopic methods, to our knowledge, a direct observation of vibrational relaxation dynamics was yet not reported. On the other hand, for **C1**, vibrational relaxation processes could not be fully resolved in ultrafast UV–VIS probe experiments.^19^ For both complexes **C0** and **C1**, our results show clear nonexponential cooling dynamics, associated with redistribution of excess energy within the complex and dissipation into low-frequency modes. This is reflected in spectral blue shifts and narrowing of bands, with 10–20 ps.

In conclusion, ultrafast mid-IR spectroscopy together with quantum chemical calculations proves to be a readily accessible method for the investigation of transient high spin states in metal–organic iron complexes and determination of their multiplicity. Moreover, ultrafast mid-IR spectroscopy provides critical structural information about the photophysical dynamics in metal–organic iron complexes, essential for developing efficient photosynthesizing complexes.

## Data Availability

Data for this paper, including transient polarization resolved data and calculations, are available at https://box.fu-berlin.de/s/3jjmfKHFTXXNYdp.

## Supporting Information Available

Polarization resolved data, detailed vibrational dynamics in the fingerprint region, femtosecond VIS-pump/VIS-probe data, FTIR spectra, detailed results of quantum chemical calculations, and description of selected low-frequency modes (PDF)
