# Supplementary material for: Femtosecond Infrared Spectroscopy Resolving the Multiplicity of High-Spin Crossover States in Transition Metal Iron Complexes
Source: J Am Chem Soc. 2024 Mar 23;146(13):9347–55. doi: 10.1021/jacs.4c01637 (PMC10995999; doi:10.1021/jacs.4c01637)
Supplement: Supplementary file 1 — ja4c01637_si_001.pdf [file ja4c01637_si_001.pdf]

Supporting Information: Femtosecond infrared spectroscopy resolving the multiplicity of high-spin crossover states in transition metal iron complexes

Clark Zahn,*,† Mariachiara Pastore,‡ J. Luis Perez Lustres,† Philippe C. Gros,¶ Stefan Haacke,§ and Karsten Heyne*,†

†*Department of Physics, Free University Berlin, Arnimallee 14, D-14195 Berlin, Germany*

‡*Université de Lorraine, CNRS, LPCT, F-54000 Nancy, France*

¶*Université de Lorraine, CNRS, L2CM, F-54000 Nancy, France*

§ *Université de Strasbourg – CNRS, IPCMS, 67034 Strasbourg, France*

E-mail: clark.zahn@fu-berlin.de; karsten.heyne@physik.fu-berlin.de

Phone: +49 30 838 56107

Anisotropic Signal

Polarisation resolved mid-IR absorption spectra averaged for delay times between 10 ps–20 ps of **C0** and **C1** are displayed in Figure S1a and S1b, respectively. Signal for parallel polarization with respect to the pump polarization is shown in black, while the signal for perpendicular polarization is shown in purple. They are almost indistinguishable, indicating that initial photoselection ist list in the first few picoseconds, or that lignands with different orientations average out the anisotropic signal. The time constant for rotational diffusion,

leading to a decay of initial photoselection is expected to be in the range of 100 ps–200 ps, so that the decay of anisotropy is expected negligible in the inspected time window.[S1] Analysis of the polarisation resolved signal shows only very small contributions for **C1** at 1590 cm^{-1} (anisotropy = 0.142) and 1440 cm^{-1}(anisotropy = 0.064). Apart from that, no significant anisotropy is found for the whole investigated spectra ranges of **C0** and **C1**. This is most likely attributed to a loss off initial photoselection of the ground state to MLCT transition, and due to the high symmetry of the ligands.

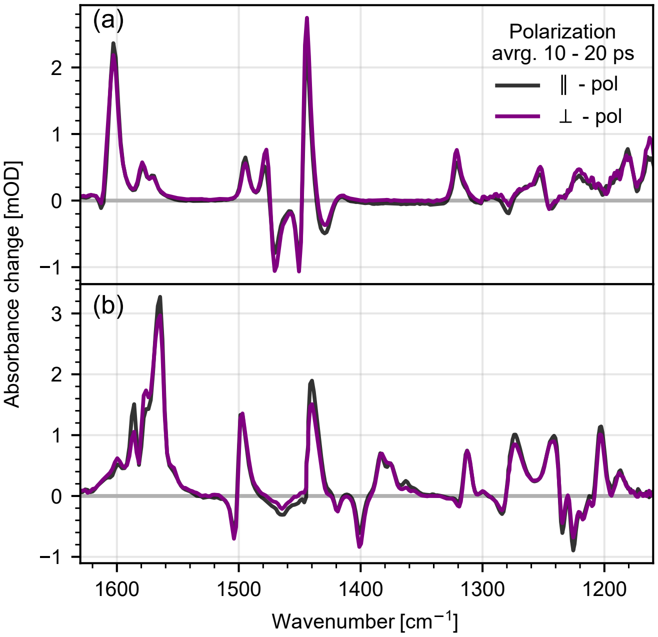

Figure S1: Transient mid-IR absorption spectra of **C0** (a) and **C1** (b) for parallel (black) and perpendicular (purple) polarization orientation, averaged from 10 ps–20 ps. Both spectra in parallel and perpendicular polarization orientation show almost identical signal strength, implicating no significant anisotropy for both **C0** and **C1**.

Transient mid-IR absorption peak shifts and detailed dynamics

Detailed dynamics of **C0** and **C1** are shown in Figure S2 and S3, respectively. Left column: transient mid-IR absorption spectra for selected delay times. Arrows indicate blue shift

of bands associated with vibrational cooling. Right column: transient traces for selected wavenumbers highlighting the spectral evolution of signal.

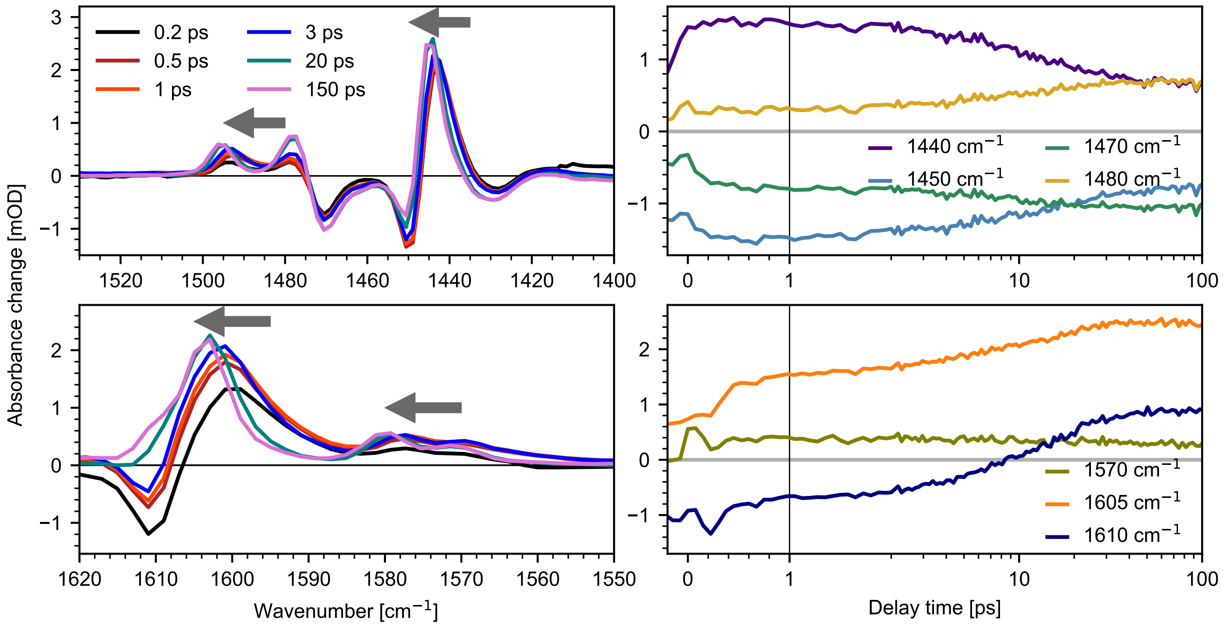

Figure S2: Detailed vibrational dynamics in the fingerprint region of **C0**. Left: Difference spectra at different delay times indicate spectral shifts due to vibrational relaxation. Right: Selected transients demonstrate the signal evolution as a function of delay time.

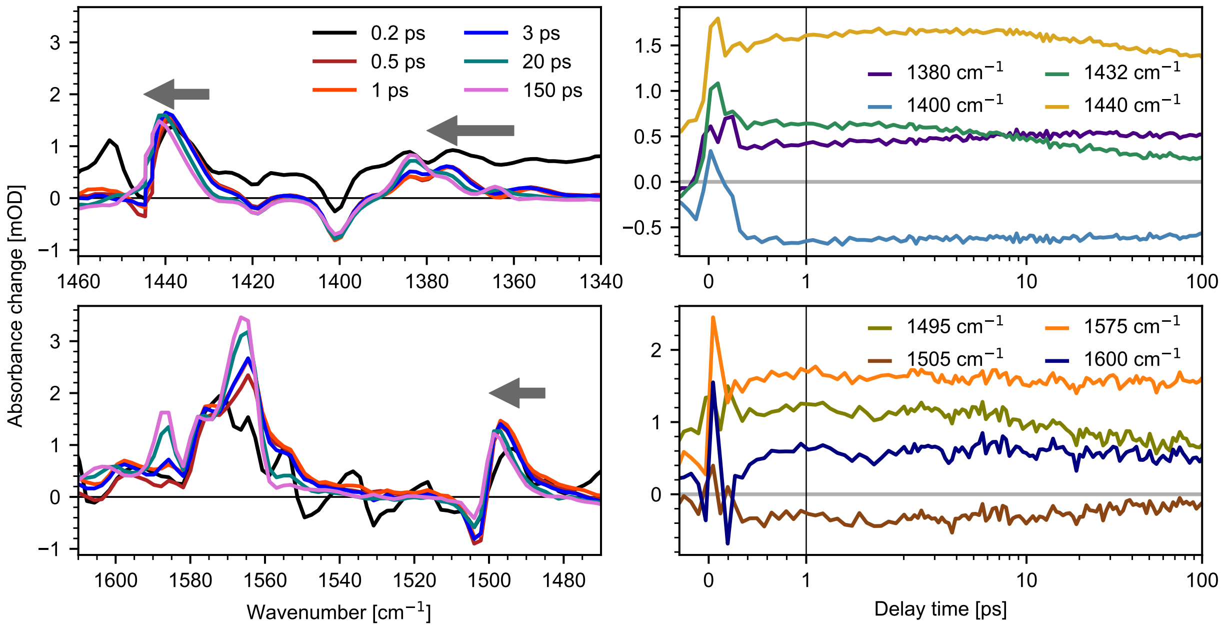

Figure S3: Detailed vibrational dynamics in the fingerprint region of **C1**. Left: Difference spectra at different delay times indicate spectral shifts due to vibrational relaxation. Right: Selected transients demonstrate the signal evolution as a function of delay time.

C1 Vis pump- Vis probe

Visible transient absorption of spectra of **C1** for selected delay times are displayed in Figure S4a. **C1** was excited at the maximum of the MLCT band at 550 nm. The spectra show an evolution of signal in the first 200 fs. Modelling of the spectral dynamics with global analysis using a single exponential decay yields a time constant of τ_1=(110±10) fs. The corresponding DAS is presented in S4b, showing a broad positive contribution spanning over almost the whole spectral range. We assign the observed dynamic to MLCT $\rightarrow$ MC deactivation with a time constant of (110 ± 10) fs. The DAS agrees well with previous work of Darari and co-workers,[S2] who investigated **C1** upon excitation at 400 nm. Different from our results, the obtained time constant of 450 fs was slower compared to our experiment when exciting **C1** at 550 nm. However, this is not surprising as the excitation at 400 nm excites a higher energetic MLCT transition.

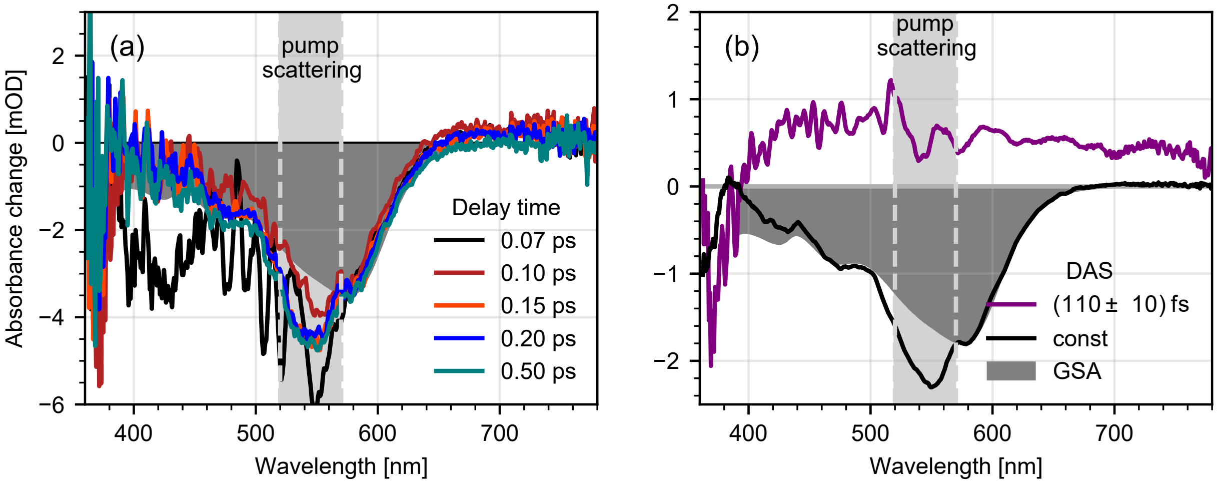

Figure S4: Transient visible absorption spectra of **C1** for selected delay times (a) and decay associated spectra (DAS) (b) obtained from global fitting the non-linear response and a single exponential decay. The time constant of (110 ± 10) fs is attributed to MLCT deactivation and MC population.

FTIR spectra

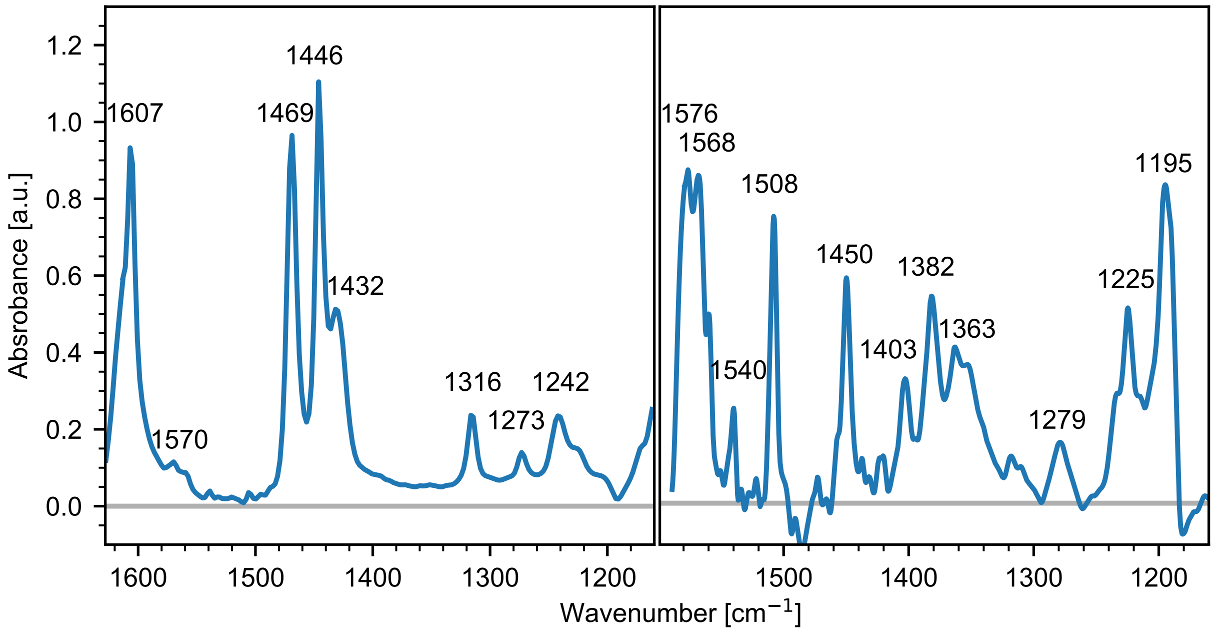

Figure S5: Ground state FTIR spectra of **C0** and **C1** in deuterated acetonitrile.

Quantum chemical calculations

Individual infrared spectra of **C0** and **C1** for different spin configurations Singlet, Triplet and Quintet are shown in Figure S6. The spectra are calculated from geometry optimization without any symmetry constraint. Infrared spectra are obtained using a Lorentzian convolution of the individual normal modes with a width of $5\,\mathrm{cm}^{-1}$. All spectra are frequency corrected with a scaling factor of 0.98, matching the experimental FT-IR spectrum in the Singlet ground state. Individual normal modes are represented with sticks. Frequency corrected spectral positions and infrared intensities of all significant bands for **C0** and **C1** are presented in Table S1 and S2, respectively. Inspection of the spectra in Figure S6 shows that a tracing of changes for individual modes is not feasible. However, accumulated frequency shifts and intensity changes of multiple modes lead to characteristic spectral changes between different multiplicities. Characterization of the different normal modes allows the following assignment: bands in the range from $1600\,\mathrm{cm}^{-1}$ to $1400\,\mathrm{cm}^{-1}$ can be associated with normal modes strongly effecting the ν(CN) and ν(CC) stretching, while the lower energetic region down to $1150\,\mathrm{cm}^{-1}$ is attributed changes of the normal modes associated with ring deformation and C-H bending modes.

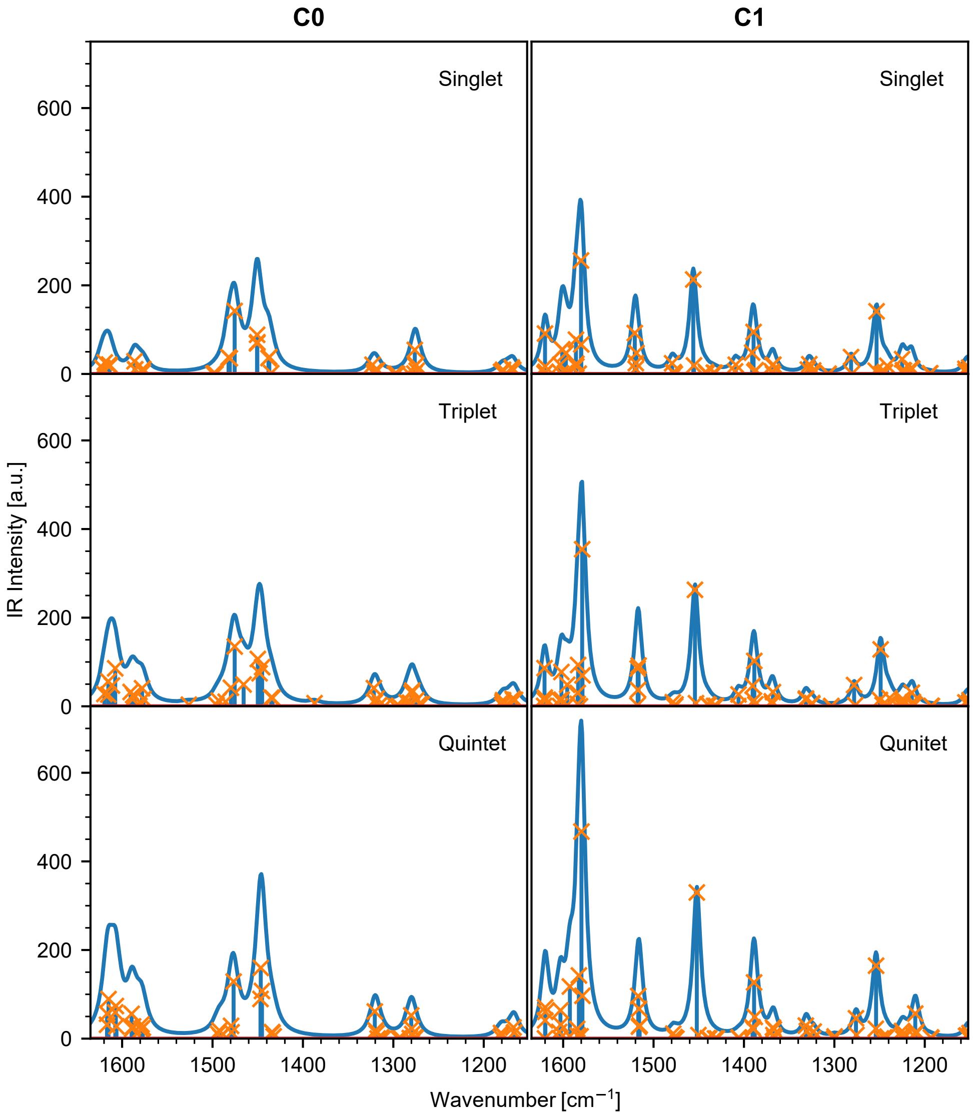

Figure S6: Calculated infrared spectra of **C0** and **C1** for different spin configurations Singlet, Triplet and Quintet. Individual normal modes are represented with sticks. The infrared intensity of each mode is highlighted with an orange x. All spectra are obtained using a Lorentzian convolution of the individual normal modes with a width of 5 cm^{-1}.

Singlet (GS)		Triplet		Quintet	
Freq [cm^{-1}]	IR intensity	Freq [cm^{-1}]	IR intensity	Freq [cm^{-1}]	IR intensity
1167.4	9.1	1165.4	14.5	1166.0	13.8
1168.0	10.0	1167.2	13.5	1166.6	13.5
1169.0	14.7	1168.1	17.0	1167.1	25.3

1178.7	9.3	1178.7	13.6	1179.9	9.4
1179.2	4.7	1179.3	9.5	1180.9	12.8
1180.8	4.8	1179.7	2.1	1181.4	3.4
1274.2	7.1	1268.2	4.8	1275.4	1.5
1274.7	20.3	1271.1	5.7	1276.6	2.6
1275.6	18.3	1274.6	13.8	1279.0	10.3
1276.3	54.2	1279.0	35.4	1279.6	22.7
1318.0	7.9	1279.8	30.3	1280.2	52.4
1319.3	4.7	1281.6	8.1	1281.8	1.3
1319.7	5.5	1285.5	9.3	1313.6	3.1
1320.9	6.3	1315.4	2.5	1318.5	12.3
1321.2	1.1	1318.4	17.6	1319.6	18.7
1323.0	20.8	1320.2	9.4	1320.5	61.3
1435.1	1.8	1321.5	41.0	1432.4	5.2
1437.2	38.1	1387.1	7.0	1433.6	13.1
1438.1	35.7	1433.6	18.8	1434.2	14.8
1450.4	71.5	1435.1	19.5	1445.3	107.5
1450.7	88.4	1445.1	88.4	1446.6	159.6
1450.8	69.0	1448.0	74.6	1447.0	89.5
1475.5	142.1	1449.9	106.8	1476.7	128.8
1480.8	35.3	1465.6	49.1	1479.0	14.4
1482.3	37.4	1475.5	134.8	1479.5	28.9
1497.8	1.2	1479.7	41.9	1491.1	3.8
1576.8	11.5	1485.5	10.2	1492.5	18.4
1576.9	9.7	1493.2	13.0	1494.0	11.0
1577.0	4.0	1495.6	2.8	1577.1	24.5
1585.5	10.2	1526.2	3.6	1578.5	32.1

1585.6	10.5	1576.4	16.6	1579.2	21.0
1586.3	28.6	1577.9	40.7	1587.9	27.6
1613.9	17.8	1586.2	20.4	1589.0	22.4
1614.2	21.1	1587.7	25.2	1589.5	56.0
1614.5	20.7	1590.6	32.3	1606.3	27.7
1619.3	25.9	1607.6	85.7	1607.0	73.6
1621.7	13.5	1611.3	46.8	1607.6	59.2
1621.9	13.7	1615.2	56.2	1615.0	89.3
		1615.4	26.4	1616.6	56.2
		1619.6	27.8	1616.7	31.3

Table S1: Frequencies and infrared intensities of normal mode vibrations of **C0** obtained from quantum chemical calculations. All frequencies are scaled with a scaling factor of 0.98. Only modes with an infrared intensity >1 are listed.

Singlet (GS)		Triplet		Quintet	
Freq [cm^{-1}]	IR intensity	Freq [cm^{-1}]	IR intensity	Freq [cm^{-1}]	IR intensity
1144.2	14.7	1142.1	17.1	1141.6	9.7
1144.7	3.9	1142.5	5.2	1142.9	8.7
1150.4	5.8	1150.6	13.7	1150.5	12.5
1150.9	14.4	1150.9	6.6	1151.1	5.2
1155.0	5.1	1154.1	7.1	1153.5	7.1
1155.1	11.7	1155.1	8.7	1154.3	6.3
1193.9	1.4	1211.3	3.6	1192.6	1.3
1214.0	11.5	1213.0	9.4	1193.3	2.7
1214.2	12.8	1213.5	3.6	1209.3	15.5
1214.9	12.4	1214.3	30.7	1210.1	12.2
1215.9	11.4	1224.2	9.6	1210.5	56.5
1223.1	7.5	1224.4	16.3	1212.2	6.6
1223.7	1.4	1225.2	5.0	1222.5	5.4
1224.1	8.4	1238.8	18.6	1224.2	9.4
1225.1	33.4	1241.9	3.2	1225.1	17.1
1241.5	18.4	1246.6	2.9	1241.3	4.6
1244.0	4.4	1248.8	128.4	1243.7	2.2

1244.9	1.9	1249.6	14.2	1245.1	2.5
1253.3	141.3	1276.9	2.4	1253.9	164.6
1254.3	7.3	1278.2	47.9	1254.2	22.3
1281.5	38.0	1324.5	5.2	1275.5	8.3
1323.2	2.5	1324.6	7.2	1276.1	46.0
1323.4	7.8	1331.4	17.5	1299.7	4.9
1327.7	21.9	1331.6	15.1	1322.9	15.9
1329.3	9.9	1367.9	32.1	1330.5	17.1
1367.5	20.8	1368.6	7.7	1331.6	29.4
1368.6	19.5	1368.8	5.5	1366.2	2.2
1369.1	5.8	1369.2	11.2	1367.0	20.8
1387.0	8.5	1387.5	4.4	1367.4	7.9
1388.4	4.9	1388.5	102.4	1367.9	24.9
1389.4	94.7	1389.0	12.9	1388.1	49.1
1391.0	48.1	1390.4	46.3	1388.8	126.8
1409.1	20.7	1406.0	26.7	1389.6	21.5
1409.9	5.8	1439.0	3.1	1390.5	25.7
1432.0	2.1	1440.5	4.4	1405.6	2.5
1433.3	2.9	1453.1	6.9	1406.1	2.0
1441.4	1.1	1454.2	262.9	1450.3	8.0
1442.4	4.9	1475.8	4.3	1452.2	329.8
1455.3	19.2	1478.7	9.2	1476.4	2.6
1456.1	213.1	1516.5	91.9	1476.8	3.3
1477.8	3.9	1517.2	37.0	1478.2	11.3
1478.2	2.9	1517.5	85.3	1515.4	29.7
1479.5	24.1	1517.8	2.4	1516.0	67.3
1518.9	47.2	1578.6	69.7	1516.1	26.9
1519.6	24.5	1579.0	354.4	1516.9	96.2
1519.8	11.5	1583.4	93.2	1578.7	96.7
1520.8	92.2	1583.9	30.1	1579.8	467.3
1580.0	66.6	1584.0	5.6	1580.7	5.2
1580.2	256.0	1595.5	48.5	1582.6	142.6
1585.4	23.1	1596.2	1.2	1583.8	6.9
1585.4	17.1	1601.7	14.9	1584.5	22.1
1585.8	77.4	1601.9	77.4	1592.5	117.6
1597.0	46.7	1604.0	13.9	1592.8	5.5
1597.8	2.3	1620.2	13.1	1602.6	31.7
1601.0	55.6	1620.4	19.7	1603.0	63.7
1601.0	54.8	1620.6	86.1	1604.4	15.1

1602.4	1.4			1619.7	60.5
1603.2	20.8			1619.9	70.6
1619.5	5.0			1619.9	41.3
1619.8	17.0				
1620.0	91.2				
1620.7	1.5				

Table S2: Frequencies and infrared intensities of normal mode vibrations of **C1** obtained from quantum chemical calculations. All frequencies are scaled with a scaling factor of 0.98. Only modes with an infrared intensity >1 are listed.

Low-frequency modes

Possible low frequency modes associated with coherent oscillations in **C1** are presented in Figure S7 and S8. Arrows indicate the atomic displacement for the respective mode.

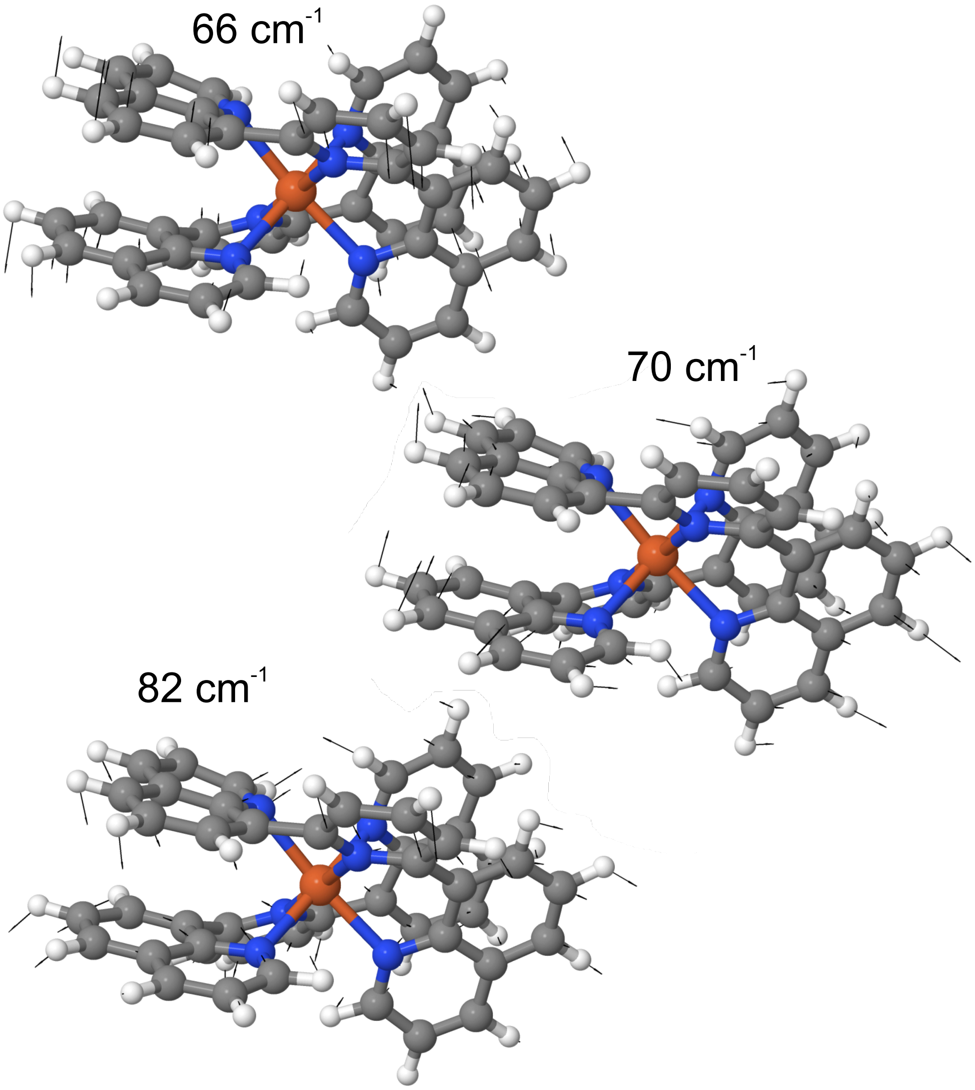

Figure S7: Calculated low frequency modes of **C1**.

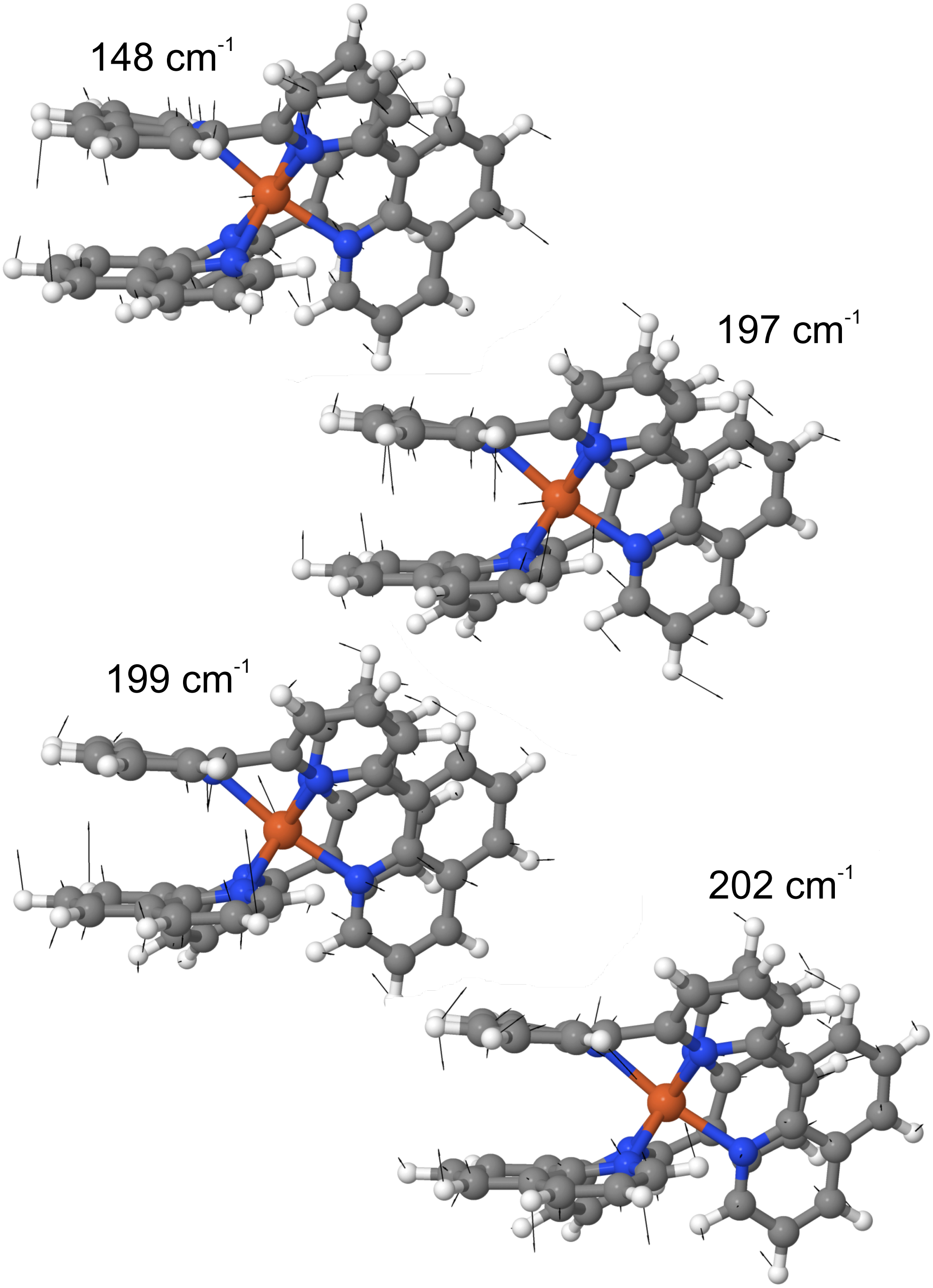

Figure S8: Calculated low frequency modes of **C1**.

References

(S1) Zahn, C.; Stensitzki, T.; Heyne, K. Femtosecond anisotropy excitation spectroscopy to disentangle the Qx and Qy absorption in chlorophyll a. *Chem. Sci.* **2022**, *13*, 12426–12432.

(S2) Darari, M.; Francés-Monerris, A.; Marekha, B.; Doudouh, A.; Wenger, E.; Monari, A.; Haacke, S.; Gros, P. C. Towards Iron(II) Complexes with Octahedral Geometry: Synthesis, Structure and Photophysical Properties. *Molecules* **2020**, *25*, 5991.
